# Pilot Study Comparing Emergency Physician and Artificial Intelligence-supported Interpretations of Electrocardiograms

**DOI:** 10.5811/westjem.48718

**Published:** 2026-04-02

**Authors:** Mehmet Gün

**Affiliations:** Maltepe University Faculty of Medicine, Department of Emergency Medicine, Istanbul, Türkiye

## Abstract

**Background:**

Artificial intelligence (AI) tools are increasingly being explored for medical applications; however, their effectiveness in emergency electrocardiogram (ECG) interpretation is under-investigated. In this pilot study we aimed to evaluate the diagnostic performance of AI interpretation of ECGs by comparing its results to those of an experienced emergency physician.

**Methods:**

We sourced 20 ECG cases representing common critical conditions from publicly available academic repositories—ST-elevation myocardial infarction, non-ST-elevation myocardial infarction, atrial fibrillation, supraventricular tachycardia, ventricular tachycardia, third-degree atrioventricular block, left and right bundle branch block, hyperkalemia, Brugada syndrome, Wellens syndrome, fusion beats, and torsades de pointes.The AI tool ChatGPT-4 and an experienced emergency physician, who served as the reference (gold standard) interpreter, independently analyzed each case across five key parameters: rhythm and heart rate; cardiac axis; ST/T segment changes; preliminary diagnosis; and emergency management recommendation. Full concordance was defined as complete agreement across all five parameters.

**Results:**

Agreement between AI and the emergency physician was observed in 18 of 20 cases (90%). The Cohen kappa was 0.80, indicating substantial chance-corrected agreement. Concordance by diagnostic category was as follows: myocardial infarction (6/6, 100%); arrhythmias including atrial fibrillation, supraventricular tachycardia, and ventricular tachycardia (6/6, 100%); conduction disorders (3/3, 100%); and hyperkalemia (1/1, 100%). Among the atypical or complex ECGs, concordance was 1/1 (100%) for Brugada syndrome, 1/1 (100%) for Wellens syndrome, 0/1 (0%) for fusion beats, and 0/1 (0%) for torsades de pointes. In the torsades case, ChatGPT did not recommend intravenous magnesium sulfate—the standard first-line treatment—despite recognizing the condition.

**Conclusion:**

An AI tool demonstrated moderate diagnostic concordance with one experienced emergency physician in interpreting some common ECG findings in the emergency setting. However, discrepancies, particularly in complex cases and critical management recommendations, highlight the need for larger scale investigations. Our findings from this pilot study do not support the independent use of AI for definitive ECG interpretation or emergency management decisions; it should serve as an adjunct tool that enhances rather than supplants human clinical judgment.

## INTRODUCTION

Interpreting electrocardiograms (ECG) in the emergency department (ED) is a standard yet critical task, as errors can yield severe repercussions. In recent years, artificial intelligence (AI) techniques have been integrated into healthcare workflows as diagnostic support systems. In emergency practice, the role of AI is swiftly broadening to include ECG interpretation, radiograph analysis, computed tomography evaluation, ultrasound image assessment, patient triage, and clinical documentation—all of which are essential for prompt and precise patient treatment.^1^,^2^ Artificial intelligence has primarily been evaluated in educational or theoretical settings. Nonetheless, its dependability in real-time emergency care remains ambiguous.^3^,^4^

Despite conjecture that AI tools could ultimately compete with or supplant human physicians in some diagnostic procedures, emergency treatment necessitates swift judgment, adaptive reasoning, and situational decision-making factors that may constrain the effective utility of existing AI systems. The potential of AI technology to assist with essential procedures, such as ECG interpretation in these contexts, remains questionable.^5^,^6^ The ECG is a rapid, readily available, and economical diagnostic instrument in the ED, essential for identifying acute coronary syndrome, arrhythmia, conduction abnormalities, and electrolyte imbalances. Notwithstanding its importance, the accuracy of interpretation may differ across clinicians due to their workload and experience.^7^

Beyond basic diagnoses, AI is being progressively employed in the ED to forecast patient deterioration, enhance bed allocation, and assist with administrative functions. This extensive application underscores its increasing significance for emergency care.^8^ Most current research on the AI tool ChatGPT (OpenAI, San Francisco, CA) has concentrated on factual inquiries or theoretical clinical scenarios, with less examination of its decision-making efficacy in real-world environments.^9^ In this study we aimed to evaluate the diagnostic accuracy and clinical significance of ECG interpretations produced by ChatGPT in comparison to those of a seasoned emergency physician, using diverse scenarios that reflect routine emergency practice.

## METHODS

This study was designed as a pilot comparative diagnostic analysis to assess the ECG interpretation capabilities of ChatGPT-4 compared to those of an experienced emergency physician. We selected 20 theoretical ECGs from two academically certified, publicly accessible repositories (ECGpedia and The ECG Library)^10^,^11^ based on their relevance to emergency practice. These cases represent an array of patterns commonly observed in the ED, encompassing arrhythmias, ischemic alterations, conduction blocks, electrolyte imbalances, and life-threatening rhythms. Specifically, the dataset included the following:

- 6 cases of myocardial infarction (ST elevation myocardial infarction/(STEMI) non-STEMI)
- 6 arrhythmias (eg, atrial fibrillation, supraventricular tachycardia, ventricular tachycardia)
- 3 conduction disorders (third-degree arterioventricular block, left bundle branch block, right bundle branch block)
- 1 electrolyte abnormality (hyperkalemia)
- 4 atypical or complex cases (Brugada syndrome, Wellens syndrome, fusion beats, torsades de pointes).

**Population Health Research Capsule**

What do we already know about this issue?

*Artificial intelligence tools like ChatGPT are being explored for medical use, but their reliability in emergency ECG interpretation remains uncertain*.

What was the research question?

*How does ChatGPT’s ECG interpretation performance compare to that of an experienced emergency physician?*

What was the major finding of the study?

*We observed 90% (18/20) concordance, with substantial agreement measured by the Cohen kappa (κ = 0.80)*.

How does this improve population health?

*AI shows promise as an adjunct tool for ECG interpretation, potentially reducing delays in emergency settings when expert consultation is not available*.

Both ChatGPT and the emergency physician independently analyzed each ECG. The emergency physician’s interpretation served as the reference standard for comparison.

The ECG images were processed using ChatGPT directly, without any instructions or context. In each instance, ChatGPT was requested to assess five standardized diagnostic criteria: 1) rhythm and heart rate; 2) cardiac axis; 3) ST/T segment changes; 4) preliminary diagnosis; and 5) emergency management recommendation. The expert emergency physician in this study was board-certified with approximately 10 years of clinical experience. In addition to structured ECG training during residency, he has served as an instructor in ECG interpretation at various congresses and educational meetings. He currently works in high-volume EDs and interprets ECGs routinely in daily clinical practice. This level of expertise provided a reliable benchmark for comparison with AI-generated outputs. He visually analyzed each ECG without access to supplementary information. All responses produced by ChatGPT were preserved in their unaltered state. The study employed solely anonymized, publicly accessible theoretical ECG cases and did not incorporate actual patient data; therefore, it was deemed exempt from institutional review board approval.

Upon the completion of both evaluation sets, interpretations were compared individually for each case. We categorized cases exhibiting agreement across all five characteristics as having full concordance. Inconsistencies were further analyzed in the discussion section. Due to the characteristics of emergency medicine training, an experienced emergency physician is anticipated to be adept at interpreting and managing the types of ECG presented in this study, possessing extensive expertise and training in acute cardiac treatment. Additionally, all AI-generated responses were verified against the original case explanations and expert diagnoses from the source websites to guarantee consistency and diagnostic accuracy.

### Electrocardiogram Data Acquisition and Preparation

The ECG images selected for each case were of high quality, with diagnostic features clearly visible. We chose these images carefully to ensure the accuracy and reliability of the assessments generated by the AI model. All ECGs were retrieved from publicly available educational platforms in web-compatible image formats (JPEG), as commonly presented for academic and clinical reference. The images were then directly uploaded to the ChatGPT interface for analysis by the GPT-4 model. No patient-identifying data or clinical metadata were used in the analysis.

### Statistical Analysis

We calculated diagnostic concordance rates for each ECG category. To quantify the statistical reliability of the observed proportions, 95% confidence intervals were computed using the Wilson score method. This method was chosen due to its balanced performance and suitability for clinical diagnostic research, as it provides more accurate interval estimates for binomial proportions, especially in studies with small sample sizes. We quantified inter-rater agreement between ChatGPT and the emergency physician using the Cohen kappa coefficient (κ) as a chance-corrected measure of concordance, complementing the percentage agreement in this pilot sample. The reporting of this diagnostic accuracy study adheres to the Standards for Reporting Diagnostic Accuracy Studies (STARD) 2015 guidelines. These guidelines ensure comprehensive and transparent reporting of methodology and results, enhancing the study’s reproducibility and generalizability. A completed STARD checklist is provided (Supplement File).

## RESULT

Overall agreement, defined as total concordance across all five diagnostic parameters, was observed in 18 of 20 ECG cases (90%). The Cohen kappa was 0.80, indicating substantial chance-corrected agreement; however, due to the pilot nature of the study and its small sample size, the overall diagnostic concordance should be interpreted with caution. The two discordant cases involved complex ECG findings: one with fusion beats, and another with polymorphic ventricular tachycardia (torsades de pointes). In the latter, ChatGPT failed to recommend intravenous magnesium sulfate as the first-line treatment, highlighting both diagnostic and therapeutic discrepancies. The table summarizes concordance across ECG categories.

**Figure 2:**
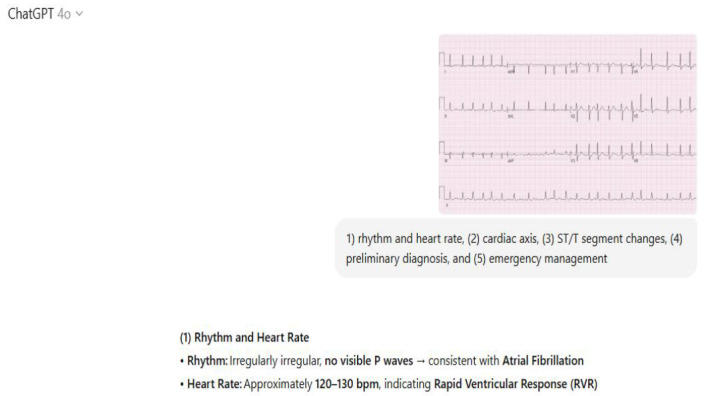
ChatGPT interpretation of an electrocardiogram demonstrating atrial fibrillation. Figures 2a and 2b highlight the model’s identification of an irregularly irregular rhythm and the absence of visible P waves. The AI tool also proposed appropriate emergency management, including rate control, anticoagulation assessment, and consideration of cardioversion if clinically indicated. This interpretation was consistent with the emergency physician’s assessment.

The AI interpretations for three ECG cases assessed in this work are depicted in Figures 1, 2, and 3 to demonstrate the model’s interpretative methodology.

Figures 3a and 3b illustrate the model’s detailed analysis, including recognition of a wide-complex tachycardia with rapid ventricular rate, extreme axis deviation, and absent discernible P waves. The AI model erroneously described the rhythm as monomorphic ventricular tachycardia rather than torsades de pointes, and it failed to suggest intravenous magnesium sulfate, the standard first-line therapy for this condition. Instead, it proposed a generalized ventricular tachycardia management protocol. This case reflects both a diagnostic and therapeutic discrepancy, underscoring the model’s current limitations in managing rhythm-specific emergencies.

## DISCUSSION

In this study we assessed the diagnostic precision and clinical applicability of ChatGPT-4, an AI-driven language model, by analyzing ECGs frequently found in emergency contexts. The model exhibited a notable level of diagnostic agreement, achieving 90% concordance with a seasoned emergency physician across various clinical presentations, including STEMI, atrial fibrillation, supraventricular tachycardia, Brugada syndrome, third-degree atrioventricular block, and electrolyte disturbances such as hyperkalemia. Zaboli et al^12^ assessed ChatGPT’s efficacy in ECG interpretation in the ED, reporting greater diagnosis accuracy for fundamental arrhythmias, but they observed diminished consistency in complex or high-risk scenarios. Recent research also explores AI’s role in improving acute myocardial infarction diagnosis. For instance, the Rule-Out Acute Myocardial Infarction Using Artificial Intelligence Electrocardiogram Analysis (ROMIAE) multicentre study by Min Sung Lee et al reported that AI-powered ECG (AI-ECG) had an area under the receiver operating curve (AUROC) of 0.878 for ruling out acute myocardial infarction, achieving 99% sensitivity for safely identifying low-risk patients and 90% specificity for high-risk patients. They also reported an AUROC of 0.951 for detecting STEMI in earlier validations. This aligns with our own study, where ChatGPT-4 also demonstrated agreement in identifying STEMI. These findings suggest that AI-driven systems may contribute to diagnostic consistency and could support care in urgent situations, especially when expert physicians aren’t immediately available.^13^ Similarly, Palermi et al have discussed the potential of AI in shaping a modern renaissance for electrocardiography, underscoring its pivotal role in advancing diagnostic capabilities.^14^

Agreement was more consistent in common emergency ECG patterns such as STEMI, atrial fibrillation, supraventricular tachycardia, Brugada syndrome, third-degree atrioventricular block, and hyperkalemia. In these scenarios, ChatGPT-4 generally identified key features and provided management suggestions aligned with emergency medicine principles. In this study, the two discordant cases—fusion beats and torsades de pointes—were those involving non-standard or morphologically complex patterns, supporting the trend of lower agreement in such categories.^15^,^16^ These discordant cases appeared to stem from the model’s difficulty in interpreting overlapping or non-standard waveform morphologies. Fusion beats require distinguishing simultaneous atrial and ventricular depolarization, a pattern that may not be easily captured through text-based pattern reasoning. Similarly, in torsades de pointes, the model did not prioritize IVintravenous magnesium sulfate as first-line therapy, indicating limitations in integrating rhythm-specific management principles.

One observed advantage was the AI model’s ability to recognize subtle ECG patterns. ChatGPT-4 correctly identified features such as the biphasic T waves associated with Wellens syndrome type B, a finding that can be challenging for non-specialist clinicians. These observations are consistent with findings by Harskamp and De Clercq,^17^ who demonstrated ChatGPT’s potential in evaluating cardiac symptoms and supporting clinical reasoning. Similarly, prior work on AI-assisted blood gas interpretation reported notable clinical alignment, highlighting the potential of AI as a supportive tool in emergency diagnostics.^18^

Nonetheless, the current investigation revealed deficiencies in AI performance. Minor errors were observed, especially in the interpretation of infrequent or intricate patterns such as torsades de pointes and fusion beats. Although ChatGPT-4 accurately identified the architecture of polymorphic ventricular tachycardia, it did not discern key characteristics such as QT interval prolongation and the dynamic changes in waveforms. Moreover, it did not recommend IV magnesium sulfate, the primary treatment for torsades de pointes, but rather suggested a broad approach for managing ventricular tachycardia. These limitations highlight the model’s present deficiency in addressing nuanced or diagnosis-dependent arrhythmias and align with the overarching difficulties encountered by AI models in understanding unusual or morphologically nuanced clinical data, underscoring the necessity for ongoing model enhancement and exposure to a more varied array of training cases.^19^,^20^

In addition to their potential clinical applications, AI models such as ChatGPT may serve as supportive tools in medical education. They can assist junior physicians and medical trainees by offering prompt feedback, improving pattern identification, and facilitating systematic diagnostic reasoning in emergency situations. Recent research has also explored how AI technologies might contribute to learning environments and support diagnostic decision-making among less experienced practitioners.^12^,^21^

This work has practical implications for the prospective incorporation of AI-driven decision-support systems into ED operations. As of March 2025, the U.S. Food and Drug Administration (FDA) had approved 1,018 AI-enabled medical devices, including 104 designated for cardiovascular medicine. The growth of FDA-sanctioned AI technology is expected to continue, potentially supporting improvements in clinical decision-making and patient care.^18^,^22^ Moreover, integrating AI algorithms directly into ECG devices could offer real-time preliminary interpretations, potentially aiding patient care and reducing diagnostic delays. This method corresponds with the extensive use of AI in clinical medicine, focused on enhancing healthcare efficiency and patient outcomes.^23^ The emergence of multimodal large-language models, exemplified by ECG-LM developed by Yang et al, which can directly read raw ECG signals and amalgamate natural language with ECG data, underscores the prospective improvements in this domain. Integrated systems have the capacity to strengthen clinical decision-support mechanisms, potentially contributing to diagnostic precision and patient outcomes, especially in settings such as the ED.^24^

## LIMITATIONS

This study has several limitations. The research was first conducted exclusively on theoretical ECG images, lacking access to clinical data, including patient history, vital signs, or laboratory results. In real emergency practice, ECG interpretation is incorporated within a wider clinical context. Additionally, the interpretations were evaluated against those of a sole emergency physician, potentially introducing individual bias and constraining generalizability. Third, while the study encompassed a broad spectrum of prevalent situations, it excluded unusual or hereditary diseases, including arrhythmogenic right ventricular cardiomyopathy. Subsequent research should incorporate real-world patient data, multicenter collaboration, and assessment of AI integration into clinical workflows to more effectively validate and enhance these first findings.

## CONCLUSION

The AI model ChatGPT-4 exhibited notable diagnostic agreement and clinical relevance in interpreting commonly encountered emergency ECG scenarios. Its ability to identify both common and subtle findings highlights its potential as a supportive decision-making tool in emergency medicine. Although minor inconsistencies were noted in rare and complex patterns, its overall consistent clinical suggestions reflect promising practical utility. The incorporation of AI tools into emergency department workflows may enhance diagnostic consistency, support physician confidence, and improve workflow efficiency. Artificial intelligence is best positioned to augment—not replace—human clinical judgment. Further validation through studies involving real-world patient data and prospective multicenter designs is essential to confirm these results and support safe clinical implementation.

## Supplementary Information



## Figures and Tables

**Figure 1:**
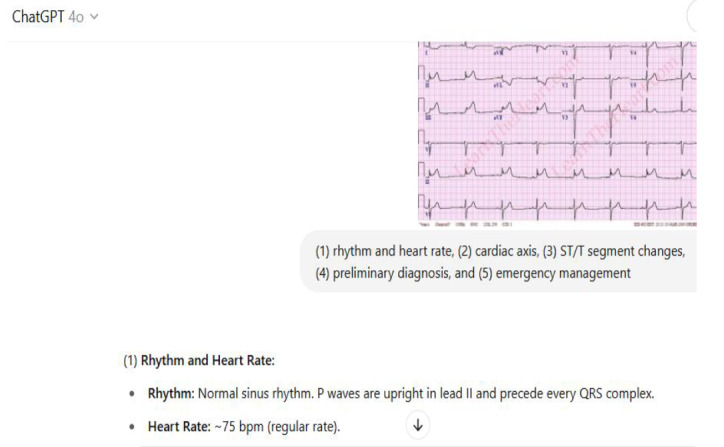
ChatGPT-4 interpretation of an electrocardiogram demonstrating an acute inferior ST-elevation myocardial infarction. Figures 1a and 1b illustrate the model’s detailed analysis, including identification of sinus rhythm, normal axis, ST elevations in leads II, III, and aVF, and reciprocal changes in aVL and V1–V2. The artificial intelligence tool also proposed appropriate emergency management in accordance with clinical standards consistent with the emergency physician’s assessment.

**Figure 1b:**
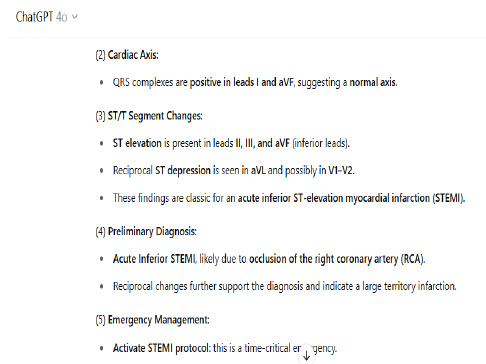
Initial portion of ChatGPT interpretation of the same electrocardiogram.

**Figure 3:**
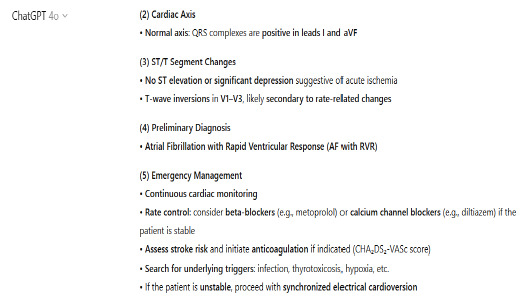
ChatGPT-4 interpretation of an electrocardiogram demonstrating polymorphic ventricular tachycardia (torsades de pointes).

**Figure 3a:**
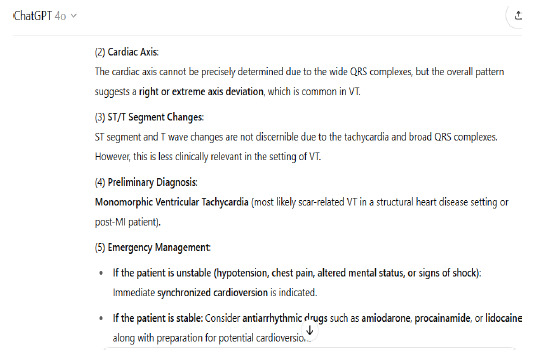
Polymorphic ventricular tachycardia (torsades de pointes) electrocardiogram provided to ChatGPT as the input prompt.

**Figure 3b:**
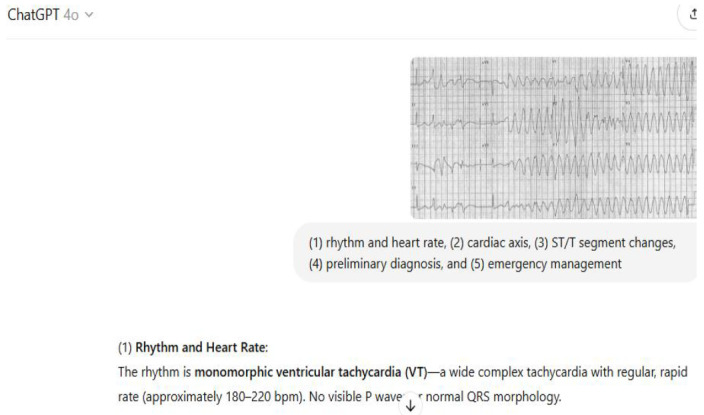
Initial portion of ChatGPT-4 interpretation for the same torsades de pointes electrocardiogram.

**Table 1:** Diagnostic concordance between the artificial intelligence tool ChatGPT-4 and an emergency physician in a study of electrocardiogram interpretation.

ECG category	Number of cases	Full concordance (n)	Concordance rate (%)	95% CI	Comments
STEMI / NSTEMI	6	6	100.0	61.0% – 100%	Consistent agreement; CI highlights variability from small sample size.
Arrhythmias (AF, SVT, VT)	6	6	100.0	61.0% – 100%	Strong agreement observed; CI suggests sample size caution.
Electrolyte abnormality	1	1	100.0	20.7% – 100%	Agreement observed; wide CI due to very small sample.
Conduction disorders	3	3	100.0	43.9% – 100%	Consistent agreement; CI reflects limited cases.
Complex atypical cases	4	2	50.0	15.0% – 85.0%	Moderate concordance; wide CI from small sample, 50% rate.
Total	20	18	90.0	68.7% – 98.2%	Overall moderate concordance, with the CI reflecting the study’s pilot nature and sample size.

*AF*, atrial fibrillation; *AV*, atrioventricular*; ECG*, electrocardiogram; *STEMI*, ST-elevation myocardial infarction; *NSTEMI*, non-ST elevation myocardial infarction; *SVT*, supraventricular tachycardia; *VT*, ventricular tachycardia.
