# Supplementary material for: Pilot Study Comparing Emergency Physician and Artificial Intelligence-supported Interpretations of Electrocardiograms
Source: West J Emerg Med. 2026 Apr 2;27(3):597–604. doi: 10.5811/westjem.48718 (PMC13246173; doi:10.5811/westjem.48718)
Supplement: Supplementary file 1 [file wjem-27-597-s001.pdf]

STARD 2015 Checklist for Diagnostic Accuracy Studies

1. 1. Identification as a study of diagnostic accuracy using at least one measure of accuracy (such as sensitivity, specificity, predictive values, or AUC).

2. 2. Structured summary of study design, methods, results, and conclusions (for abstract).

Response: Structured abstract provided with background, methods, results, and conclusions including diagnostic accuracy statistics.

Response: Comparative diagnostic accuracy study using retrospective ECG cases.

Response: 18/20 concordant cases; detailed results reported by ECG category in a table with CI.

3. 3. Scientific and clinical background, including the intended use and clinical role of the index test.

4. 4. Study objectives and hypotheses.

5. 5. Whether data collection was planned before the index test and reference standard were performed (prospective) or after (retrospective).

6. 6. Eligibility criteria.

7. 7. On what basis potentially eligible participants were identified (such as symptoms, results from previous tests, inclusion in registry).

Response: 20 theoretical ECG cases sourced from ECGpedia and ECG Library, commonly seen in emergency practice.

Response: 18/20 concordant cases; detailed results reported by ECG category in a table with CI.

8. 8. Where and when potentially eligible participants were identified (setting, location and dates).

Response: 20 theoretical ECG cases sourced from ECGpedia and ECG Library, commonly seen in emergency practice.

9. 9. Whether participants formed a consecutive, random or convenience series.

Response: 20 theoretical ECG cases sourced from ECGpedia and ECG Library, commonly seen in emergency practice.

10. 10. Index test, in sufficient detail to allow replication.

11. 11. Reference standard, in sufficient detail to allow replication.

12. 12. Rationale for choosing the reference standard (if alternatives exist).

13. 13. Definition of and rationale for test positivity cut-offs or result categories of the index test, distinguishing pre-specified from exploratory.

14. 14. Definition of and rationale for test positivity cut-offs or result categories of the reference standard, distinguishing pre-specified from exploratory.

15. 15. Whether clinical information and reference standard results were available to the performers/readers of the index test.

Response: 18/20 concordant cases; detailed results reported by ECG category in a table with CI.

16. 16. Whether clinical information and index test results were available to the assessors of the reference standard.

Response: 18/20 concordant cases; detailed results reported by ECG category in a table with CI.

17. 17. Methods for estimating or comparing measures of diagnostic accuracy.

18. 18. How indeterminate index test or reference standard results were handled.

Response: 18/20 concordant cases; detailed results reported by ECG category in a table with CI.

19. 19. How missing data on the index test and reference standard were handled.

20. 20. Any analyses of variability in diagnostic accuracy, distinguishing pre-specified from exploratory.

21. 21. Intended sample size and how it was determined.

22. 22. Flow of participants, using a diagram.

Response: 20 theoretical ECG cases sourced from ECGpedia and ECG Library, commonly seen in emergency practice.

23. 23. Baseline demographic and clinical characteristics of participants.

Response: 20 theoretical ECG cases sourced from ECGpedia and ECG Library, commonly seen in emergency practice.

24. 24. Distribution of severity of disease in those with the target condition.

25. 25. Distribution of alternative diagnoses in those without the target condition.

26. 26. Time interval and any clinical interventions between index test and reference standard.

27. 27. Cross tabulation of the index test results (or their distribution) by the results of the reference standard.

Response: 18/20 concordant cases; detailed results reported by ECG category in a table with CI.

28. 28. Estimates of diagnostic accuracy and their precision (such as 95% confidence intervals).

29. 29. Any adverse events from performing the index test or the reference standard.

30. 30. Study limitations, including sources of potential bias, statistical uncertainty, and generalisability.
